# Digital technologies in bronchiectasis physiotherapy services: a survey of patients and physiotherapists in a UK centre

**DOI:** 10.1183/23120541.00013-2024

**Published:** 2024-10-06

**Authors:** Katherine O'Neill, Brenda O'Neill, Rebecca H. McLeese, James D. Chalmers, Jeanette Boyd, Anthony De Soyza, Paul McCallion, Judy M. Bradley

**Affiliations:** 1 Wellcome-Wolfson Institute for Experimental Medicine, Queen's University Belfast, Belfast, UK; 2 Centre for Health and Rehabilitation Technologies, Ulster University, Derry/Londonderry, UK; 3 Division of Molecular and Clinical Medicine, University of Dundee, Ninewells Hospital and Medical School, Dundee, UK; 4 European Lung Foundation, Sheffield, UK; 5 Freeman Hospital Newcastle and Newcastle University, Newcastle upon Tyne, UK

## Abstract

**Introduction:**

We aimed to explore how digital technology is currently used, could be used and how services could be improved in order to optimise bronchiectasis physiotherapy care.

**Methods:**

Online surveys were designed and distributed amongst people with bronchiectasis and physiotherapists in Northern Ireland. Responses to closed and open question formats were collected and analysed.

**Results:**

The survey was completed by 48 out of 100 physiotherapists (48%) between January 2020 and January 2021 and by 205 out of 398 people with bronchiectasis (52%) between October 2020 and October 2021. 56% of physiotherapists (27 out of 48) reporting using some type of digital technology to facilitate services, whereas 44% (21 out of 48) reported that they had never used a digital technology in this patient group. When physiotherapists were asked whether they would be likely to use certain remote and/or digital options to deliver follow-up care for airway clearance techniques, most (31–38 out of 48; 65–79%) indicated that they would. Regarding patient responses, most reported that they would use telephone consultation (145 out of 199, 73%) and a smaller proportion were likely to use video consultation (64 out of 199, 32%). The most commonly mentioned theme for improvement amongst patients was follow-ups, while improved access, quality of services and treatments were the most commonly mentioned amongst physiotherapists.

**Conclusion:**

Despite a large proportion of physiotherapists in this survey reporting no current use of digital technology in bronchiectasis physiotherapy care, there was significant interest and willingness to do so, amongst both physiotherapists and patients. This survey highlighted a range of care areas, specifically follow-up visits, where digital methods could be further explored.

## Shareable abstract

**Digital health technologies have the potential to support personalised approaches and innovative care pathways in bronchiectasis physiotherapy care** https://bit.ly/42otjRD

## Introduction

Airway clearance techniques (ACTs) are central in the management strategy for patients with bronchiectasis [1]. In our recent survey of 205 patients with bronchiectasis in Northern Ireland in the UK we described current practices for ACTs in patients with bronchiectasis from the patient and physiotherapist perspective [2]. We reported that physiotherapists are generally following the bronchiectasis guidelines and using a stepwise approach to management. Despite being a priority, accessibility and implementation of ACTs are variable in clinical settings [3]. There is a need to provide more accessible care, deliver education and optimise implementation of ACTs in bronchiectasis physiotherapy services.

Increasingly, the role of technology in boosting effectiveness of ACTs and, more broadly, respiratory physiotherapy management is being explored [4]. In the current healthcare environment, digital technologies are widely promoted as contributing to accessible, efficient and patient-centred care [5]. The behaviours of healthcare professionals and patients have changed owing to the pandemic, with patients accessing services in new ways [6]. Moves to digital and remote methods to deliver care have already been reported in a range of chronic respiratory diseases across a breadth of clinical management areas, *i.e.* telerehabilitation [7], remote consultations [4], home monitoring [8], home management and monitoring treatment adherence [4]. Specifically in bronchiectasis, Congrete and Metersky [9] detailed their experience of telemedicine and remote monitoring in a commentary including these areas of management. In addition, home spirometry and home monitoring of physical activity and quality of life were reported. In this commentary, the authors provided a framework to inform the tailored use of components of telemedicine to individuals, based on the evidence and availability of the necessary technologies. Physiotherapy is a central part of the main management strategy for patients with bronchiectasis [10], and clinical decision-making regarding ACT prescription can be complex [11]. It is important to consider the factors that could enable physiotherapists to use digital and remote methods in their practice. Equally, it is important to ascertain patients’ perceptions and enablers of and barriers to such methods.

## Aim

We aimed to explore how digital technology is currently used (physiotherapy survey) and could be used (patient and physiotherapy surveys), and how services could be improved to optimise care (patient and physiotherapy survey).

## Methods

Patient and physiotherapist online surveys were designed, distributed, collected and analysed as previously described [2]. Responses were sought *via* closed and open question formats. For ranking questions, mean rank order of factors were summarised. Responses to free text questions were analysed and themes of common or repeated features of participants’ qualitative responses were categorised, first by two researchers independently who then met and agreed final themes.

## Results

As previously reported, between January 2020 and January 2021, the online physiotherapist survey (supplement 1) was sent to 100 physiotherapists identified as therapists with a link to bronchiectasis or respiratory services. The survey was completed by 48 out of 100 physiotherapists (48%) from five sites in Northern Ireland. Respondents ranged in level of seniority from newly qualified to senior specialised physiotherapist and were based across a range of settings (52% (25 out of 48) hospital based and 48% (23 out of 48) community based). The patient survey (supplement 2) was distributed to 398 patients with bronchiectasis from the Bronch-UK/EMBARC Registry and the survey was completed by 205 individuals (52%) between October 2020 and October 2021 [1]. The total number of responses to each question is noted herein. Whilst the main results from this survey were reported in a separate publication, the specific survey question results reported in this paper have not been previously reported.

### Current use of digital technology

**FIGURE 1:**
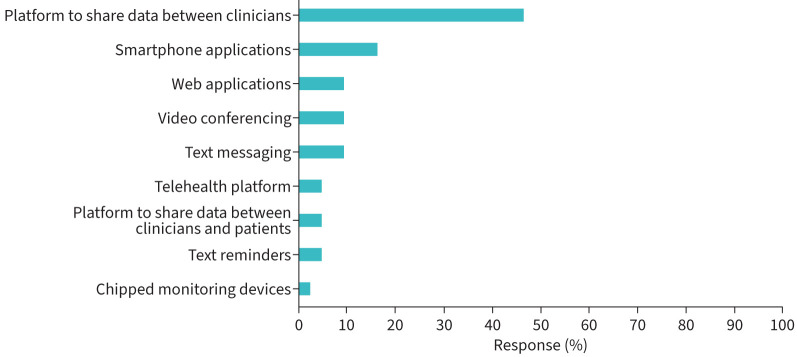
Forms of digital technology used by physiotherapists in patients with bronchiectasis (n=43).

56% of responding physiotherapists (27 out of 48) reporting using some type of digital technology to facilitate clinical physiotherapy services for people with bronchiectasis, where as 44% (21 out of 48) reported that they had never used a digital technology in this patient group. Of those physiotherapists that did, just under half (22 out of 48; 46%) of physiotherapy respondents reported using a platform to share data between clinicians. Smaller proportions of physiotherapist respondents reporting using a varied range of other technologies (1–8 out of 48; 2–16%) (figure 1).

**FIGURE 2:**
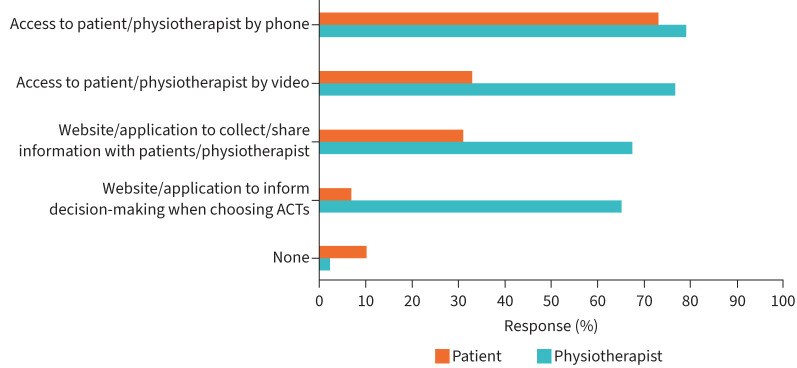
What digital or remote options would you use for follow-up (n=199 patients; n=43 physiotherapists). ACT: airway clearance technique.

### Future use of digital technology

When physiotherapists were asked whether they would be likely to use certain remote and/or digital options to deliver follow-up care for ACTs (examples provided: telephone consultation, video consultation, patient portals, clinical decision support systems), most (31–38 out of 48; 65–79%) indicated that they would (figure 2). Regarding patient responses, most reported that they would use telephone consultation (145 out of 199; 73%). A smaller proportion of patients were likely to use video consultation (64 out of 199; 32%), patient portals (61 out of 199; 31%) or clinical decision support systems (13 out of 199; 7%). 10% of patients (20 out of 199) reported that they would not use digital options, and in response to “other comments”, 23% of patients (45 out of 199) reported that they would prefer face-to-face appointments as the method of follow-up.

When considering what aspects of physiotherapy care could be facilitated or delivered using digital technologies, physiotherapists rated a range of service areas from 1 (most important) through to 6 (least important). Patient education, monitoring of patient symptoms and clinical status and monitoring of patient adherence were ranked as highest priority (ranking 2.3, 2.4 and 3.5 out of 6, respectively).

### Areas for service improvement

**FIGURE 3:**
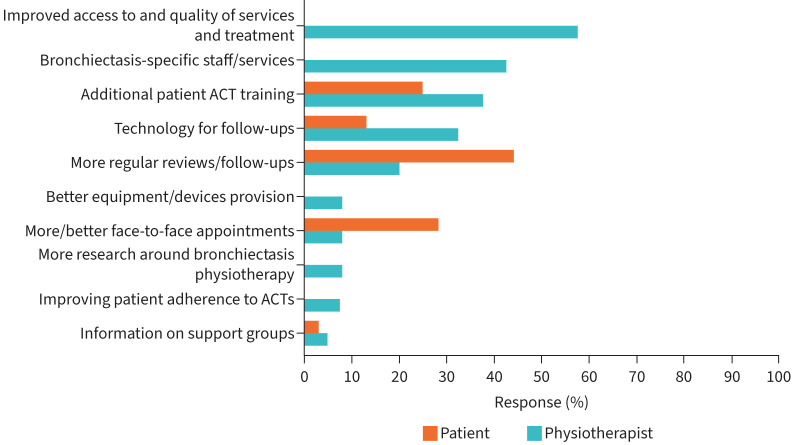
Areas of improvement (n=40 physiotherapists; n=179 patients). ACT: airway clearance technique.

Both physiotherapists (40 out of 48; 83%) and patients (179 out of 205; 87%) provided further comments on how the service offered to patients with bronchiectasis could be improved. Figure 3 presents the emergent themes. Both physiotherapists and patients highlighted patient ACT training, use of technology for follow-ups, regularity of follow-ups, regularity of face-to-face appointments and support groups as areas for improvement. Interestingly, the most commonly mentioned theme for improvement amongst patients was follow-ups whilst improved access and quality of services and treatments was the most commonly mentioned theme amongst physiotherapists.

## Discussion

Recent literature, including the European Respiratory Society statement on ACTs in adults with bronchiectasis, emphasises the importance of considering patient and physiotherapist perceptions to facilitate implementation and adherence to ACTs [1, 10]. Our survey findings report that many patients want more regular follow-up, and remote and digital methods may offer alternative ways that are acceptable to patients and physiotherapists. In alignment with these findings, a survey of bronchiectasis patients receiving remote physiotherapy consultation (telephone and video depending on preference) by McCallion *et al.* [12] found that patient satisfaction was maintained using remote methods. Use of remote methods may facilitate physiotherapy follow-up of a growing population with bronchiectasis in accordance with guidelines, as well as improving the convenience of access to care for patients with severe lung disease or, indeed, other comorbidities that impact their mobility, and for patients who live long distances from their regional centre. Based on the evidence from studies of telemedicine, remote monitoring, home monitoring of treatment, exacerbations, physical activity and telerehabilitation across chronic respiratory disease more broadly, Congrete and Metersky [9] provided a framework to facilitate tailoring of available technologies to individual need, based on the supporting evidence and availability of the necessary technologies.

In this study, most patients indicated greater interest in follow-up for physiotherapy using telephone compared with video consultation and application-based platforms, likely due to high familiarity with this method and potential lack of confidence with other forms of technology [13]. Patient–provider communication through telephone has been used previously for information, advice, reassurance or monitoring purposes in respiratory patient populations [5]. Most physiotherapy respondents indicated that they would be open to using video call methods for follow-up. Video calls could be beneficial for treatments such as nebulisers and ACTs because assessment and correction of technique can be facilitated using this method [14]. Such methods have been employed in patients with cystic fibrosis to deliver exercise interventions [15]. Physiotherapist time and capacity to employ such methods requires further careful consideration.

Physiotherapy respondents in this study also indicated a willingness to use applications or web-based platforms to share information (*e.g.* monitor patient symptoms and treatment adherence), communicate and inform decision-making. Such digital technologies could offer benefits including improved surveillance of symptoms and may assist in preventing pulmonary exacerbations. A better understanding of the relationship between symptoms and treatment adherence could facilitate clinical decision-making and reinforce to patients the importance of adhering to their treatments. In bronchiectasis specifically, understanding how to adjust ACT frequency and duration is key to the “step-up” and “step-down” management recommendations [1]. This is an area of care that could be optimised with the use of digital technologies, *e.g.* patient recommendations or alerts on increasing frequency and/or duration of ACT in response to increased symptoms recorded in a patient digital diary or application. In previous research, digital monitoring of patient adherence and self-management in respiratory populations has been reported to improve the ability of healthcare professionals to target modifiable determinants of treatment adherence with behaviour-change strategies [16]. Using digital technologies to monitor and improve ACT quality in children with cystic fibrosis has been described, highlighting the importance of prescription conformant ACTs to ensure clinical benefit. Exploring technology application in other disease areas may be important to better understand how and when ACTs will be useful [17]. Accelerated by the COVID-19 pandemic, respiratory digital monitoring is increasing and there is a need to promote sustainable implementation to achieve success within routine clinical care. Successful implementation is complex, requiring understanding of patient, professional and organisational perspectives [5]. Lack of access to digital technologies and low digital literacy are potential obstacles [16]. Despite the majority of responding physiotherapists reporting a willingness to use digital technologies in their management of people with bronchiectasis, a large proportion reported never using a digital technology. This may have been due to lack of access to technologies, lack of knowledge and low confidence levels with technology use. The level of patient and therapist digital literacy, as well as the feasibility and acceptability of any digital methods, needs to be considered ahead of implementation and to support the enhancement of digital competency generally [18]. Assessing digital literacy in patients and clinicians is important to direct appropriate education and training. Tools such as the Digital Health Readiness Questionnaire have been suggested to gain insight into the care pathway, tailor digital care pathways accordingly and offer those with low digital readiness appropriate education programmes to facilitate participation [19].

This study has a number of strengths. The ACTs used by patients surveyed in this study were similar to those reported in populations surveyed throughout the UK (Bronch-UK/EMBARC Registry), with most patients reported using active cycle of breathing technique, huffing and exercise and/or physical activity [3].

This study has a number of limitations. Results are based on patient survey data from one region in the UK; therefore, results may not be generalisable to other regions. Subgroup analysis of patient data based on location of care (hospital *versus* community care) was not possible with this data but may be important to explore to inform the design of future telehealth resources. Future studies using mixed methods involving both survey and qualitative methods, *e.g.* focus groups and interviews, should obtain more in-depth information about the patient and physiotherapist perspectives to design ACT programmes using telehealth in this population.

The sample size of the physiotherapist survey (n=48) from a specific UK area is also a limitation. Healthcare practices can vary across UK regions and therefore these results may not accurately represent the broader landscape of physiotherapy services in the UK. Furthermore, whilst this study highlights patient and therapist digital literacy as an important factor influencing uptake of digital technologies, it was not assessed.

### Conclusion

Despite a large proportion of physiotherapists in this survey reporting no current use of digital technology in their management of these patients, there was significant interest and willingness to do so, amongst both physiotherapists and patients. This survey highlighted a range of care areas, specifically follow-up visits, where digital and/or remote methods could be used. Further studies to explore the acceptability and usage of such technologies in physiotherapy practice in this patient group is warranted.

## Supplementary material

10.1183/23120541.00013-2024.Supp1 **Please note:** supplementary material is not edited by the Editorial Office, and is uploaded as it has been supplied by the author.
Patient survey version 1 00013-2024.SUPPLEMENT1
Patient survey version 2 00013-2024.SUPPLEMENT2
