# Supplementary material for: Digital technologies in bronchiectasis physiotherapy services: a survey of patients and physiotherapists in a UK centre
Source: ERJ Open Res. 2024 Oct 6;10(3):00013-2024. doi: 10.1183/23120541.00013-2024 (PMC11163277; doi:10.1183/23120541.00013-2024)
Supplement: Supplementary file 1 [file 00013-2024.SUPPLEMENT1.pdf]

Respiratory physiotherapy airway clearance techniques and services for bronchiectasis

Version 1 26/08/2019

About the study

You are being invited to participate in this survey research as you are a physiotherapist who treats people with bronchiectasis. Your participation is highly valued, and we thank you for your time. This study is being led by Queen's University Belfast (Dr Katherine O'Neill, Prof Judy Bradley) and funded by the Northern Ireland Chest Heart and Stroke.

What is this survey about?
This survey will explore airway clearance technique practice and respiratory physiotherapy services for bronchiectasis in Northern Ireland. Your information and views are very important to us as it is you who really knows how services could be improved for people with bronchiectasis. We are running a patient survey alongside this physiotherapist survey to determine what current practice is in Northern Ireland.

Who should complete this survey?
Please complete this survey if you are a physiotherapist based in Northern Ireland, who currently treats people with bronchiectasis, or has treated people with bronchiectasis in the last month. This may have been in any setting for example; outpatient, inpatient, community, domiciliary.

Do I have to take part?
Your participation in this survey research is entirely voluntary. It is up to you to decide whether or not you take part. If you participate, you can withdraw at any time by closing the page.

What will happen if I take part?
Participating involves completing an online survey which will take approximately 20 minutes. The survey questions will ask you about your current airway clearance technique practice and physiotherapy services for people with bronchiectasis.

What are the possible benefits?
While there will be no direct benefits for you as an individual by participating in this survey, we hope to use the findings to help improve physiotherapy services for people with bronchiectasis. People with bronchiectasis have said that access to chest physiotherapy is a top research priority (EMBARC consensus statement).

Confidentiality and Data Protection
The survey can be completed anonymously and your responses will not be identifiable to you. Your data will be kept confidential and will be stored securely by Queen's University Belfast and only members of the research team will have access to the information. All data stored on the Queen's University Belfast system will comply with the University's policy. Your data will remain on SurveyMonkey for 24 months. This survey has been the subject of ethical review and adheres to applicable data security standards. All data will be destroyed 5 years after completion of the study. Anonymous data collected from the survey may be transferred to various locations where SurveyMonkey has offices and shared with other organisations.

Queen's University Belfast is the sponsor for this study based in the United Kingdom. We will be using information from you in order to undertake this study and will act as the data controller for this study. This means that we are responsible for looking after your information and using it properly. Queen's University Belfast will keep identifiable information about you for 5 years after the study has finished. Your rights to access, change or move your information are limited, as we need to manage your information in specific ways in order for the research to be reliable and accurate. If you withdraw from the study, we will keep the information about you that we have already obtained. To safeguard your rights, we will use the minimum personally-identifiable information possible.
You can find out more about how we use your information.

Who can I contact if I have more questions?
You can contact the principal investigator (Prof Judy Bradley/Dr Katherine O'Neill) on ACTBEStudy@qub.ac.uk / 00 44 (0)28 9097 6005 if you have any questions or if you do not wish to be take part. Thank you for reading this information.

This survey consists of 3 sections:
Section 1: Consent
Section 2: About you
Section 3: Questions

Respiratory physiotherapy airway clearance techniques and services for bronchiectasis

Section 1: Consent Form

* 1. I understand that my participation in this survey research study is voluntary and that I am free to withdraw at any time by closing the page.

☐ Yes

* 2. I understand that anonymized data gathered in this study will be used in research publications.

☐ Yes

* 3. I understand that data collected during the study may be looked at by individuals involved in the study or from regulatory authorities, where it is relevant to my participation in this research.

☐ Yes

* 4. I understand that the data will be stored in a safe manner in Queen's University Belfast and will be destroyed after 5 years of study completion.

☐ Yes

* 5. I confirm that I have completed this survey only once.

☐ Yes

* Please enter your survey number as provided on the invitation email:

If you do not have your survey number please contact the research team on 0044 (0)28 9097 6005 or ACTBEStudy@qub.ac.uk

* 6. **(Optional)** I am willing to be contacted by the research team to help interpret the overall survey results.

If yes, Queen's University Belfast will require your name and address, or email address to facilitate contact. Please click here to provide your details (this will open a new page, please return to this page when completed).

- [ ] Yes. I have provided my contact details.
- [ ] No

Respiratory physiotherapy airway clearance techniques and services for bronchiectasis

Section 2: About you

Please answer these questions based on your usual circumstances/service.

* 1. Who is your employer in Northern Ireland?

- ◯ Belfast Health and Social Care Trust
- ◯ South Eastern Health and Social Care Trust
- ◯ Northern Health and Social Care Trust
- ◯ Southern Health and Social Care Trust
- ◯ Western Health and Social Care Trust
- ◯ Other

* 2. What is the approximate amount of time you spend in post, providing respiratory physiotherapist services to bronchiectasis patients (in a typical week)?

- ◯ 0.5 day
- ◯ 1 day
- ◯ 1.5 days
- ◯ 2.0 days
- ◯ 2.5 days
- ◯ 3.0 days
- ◯ 3.5 days
- ◯ 4.0 days
- ◯ 4.5 days
- ◯ 5.0 days
- ◯ Other

* 3. Where is your work setting? (Please select all that apply).

Please state the approximate number of days spent in this setting, providing respiratory physiotherapist services to bronchiectasis patients in a typical week in the comments box. (e.g. Hospital based: 2.5 days, Domiciliary: 2.5 days).

- ☐ Hospital based
- ☐ Health centre in the community
- ☐ Domiciliary
- ☐ Other

* Approximate number of days spent in each setting in a typical week:

* 4. What is your current Agenda for Change grade?

Respiratory physiotherapy airway clearance techniques and services for bronchiectasis

Section 3: Questions on Airway Clearance Technique practice

Please answer these questions based on your usual circumstances/service.

* 1. What factors influence your choice of a particular airway clearance technique for a person with bronchiectasis (when clinically stable)?

Please rank in order of importance, **1 being most important, 10 being least important.**

- My knowledge and experience of using the airway clearance technique
- Availability/access to the airway clearance technique
- The co-morbidities of the person with bronchiectasis
- The understanding and competence of the person with bronchiectasis
- Staff time allocated to the appointment, to see the person with bronchiectasis
- The preferences of the person with bronchiectasis for one airway clearance technique versus another
- The symptoms of the person with bronchiectasis

The disease stability of the person with bronchiectasis e.g. frequency of pulmonary exacerbations

The disease severity of the person with bronchiectasis e.g. mild, moderate, severe disease; microbiological status

Local tariffs or prescriptions/funding available for equipment

Respiratory physiotherapy airway clearance techniques and services for bronchiectasis

Please answer these questions based on your usual circumstances/service.

* 2. Think about the **first visit** when you see a person with bronchiectasis for airway clearance techniques.

What do you think are the most important parts of a **first visit** with a person with bronchiectasis for airway clearance techniques?

Please rank in order of importance, **1 being most important, 8 being least important**.

- Providing information about airway clearance techniques and why they are important
- Performing a physiotherapy chest assessment
- Teaching an airway clearance technique
- Setting a personal action plan for the airway clearance techniques with the patient (an action plan is a written plan that details the patient's symptoms and what to do when well and when not well)
- Having enough time for the first appointment
- Providing information about other physiotherapy treatments in addition to airway clearance techniques (pulmonary rehabilitation, medications for airways clearance, incontinence treatments)
- Providing information about support groups
- Providing contact details and instruction on how to access a physiotherapist in the future

Respiratory physiotherapy airway clearance techniques and services for bronchiectasis

Please answer these questions based on your usual circumstances/service.

* 3. Think about patient access to physiotherapy for their bronchiectasis.

For the **first follow-up visit** (first time the patient is seen after the initial consultation with the physiotherapist), what do you think is most important?

Please rank in order of importance, **1 being most important, 4 being least important.**

- Patient access to a physiotherapist who is specialist in bronchiectasis
- Patient access to a physiotherapist who works with respiratory patients (but not necessarily specialist in bronchiectasis)
- Patient visit in a dedicated bronchiectasis clinic
- Patient visit in a location of their choice

Respiratory physiotherapy airway clearance techniques and services for bronchiectasis

Please answer these questions based on your usual circumstances/service.

* 4. Think about follow-up of people with bronchiectasis for airway clearance techniques.

What patient factors prioritise **follow-up** of people with bronchiectasis for airway clearance techniques?

Please rank in order of importance, **1 being most important, 6 being least important.**

- The symptoms of the person with bronchiectasis
- The co-morbidities of the person with bronchiectasis
- The understanding and competence of the person with bronchiectasis with their airway clearance technique
- The disease stability of the person with bronchiectasis e.g. frequency of pulmonary exacerbations
- The disease severity of the person with bronchiectasis e.g. mild, moderate, severe disease; microbiological status
- Local tariffs or prescriptions/funding available for equipment

Respiratory physiotherapy airway clearance techniques and services for bronchiectasis

Please answer these questions based on your usual circumstances/service.

* 5. As the service is modernised, there may be new ways to follow-up people with bronchiectasis for airway clearance techniques.

Which of the following do you think you would use to follow-up people with bronchiectasis for airway clearance techniques, if they were available to you?

Please select all that apply.

- [ ] Access to the patient by phone
- [ ] Use of a video link or Skype to see and speak to the patient
- [ ] Use of a website and/or phone app to collect and share information about the patient's condition (e.g. information about symptoms, lung function) and communicate with the patient
- [ ] Use of a website and/or phone app to help inform your decision on which airway clearance techniques to do
- [ ] None
- [ ] Other (please specify)

Respiratory physiotherapy airway clearance techniques and services for bronchiectasis

Please answer these questions based on your usual circumstances/service.

* 6a. Potential digital health technologies in respiratory health include a broad range of different applications.

Have you **ever used** any of the listed forms of digital technology to facilitate **clinical** physiotherapy services for people with bronchiectasis?

Please select all that apply.

- ☐ I have never used digital technology to facilitate physiotherapy services for people with bronchiectasis
- ☐ Text messaging
- ☐ Text reminders
- ☐ Web-based applications (i.e. on the PC)
- ☐ Mobile applications (i.e. on the phone)
- ☐ Chipped devices to monitor use (i.e. inhalers, nebulisers)
- ☐ Platforms to share and exchange data between clinicians (i.e. electronic medical records)
- ☐ Platforms to share and exchange data between clinician and patient (i.e. web-based or mobile apps that share and exchange symptoms and lung function)
- ☐ Other

QUEEN'S UNIVERSITY BELFAST

Respiratory physiotherapy airway clearance techniques and services for bronchiectasis

Please answer these questions based on your usual circumstances/service.

* 6b. Please tell us more about the digital technology used.

Respiratory physiotherapy airway clearance techniques and services for bronchiectasis

Please answer these questions based on your usual circumstances/service.

* 7. In your opinion, in what areas do you think digital technology could facilitate physiotherapy services for people with bronchiectasis?

Please rank areas in order of importance, **1 being most important, 7 being least important**.

- Providing patient education on bronchiectasis physiotherapy services
- Delivering physiotherapy treatment
- Providing patient monitoring of symptoms and clinical status
- Monitoring patient adherence to physiotherapy treatment
- Delivering a patient consultation
- Facilitating physiotherapy clinical decision making
- Facilitating multi-disciplinary clinician communication

QUEEN'S UNIVERSITY BELFAST

Respiratory physiotherapy airway clearance techniques and services for bronchiectasis

Please answer these questions based on your usual circumstances/service.

* 8. How do you think we could improve the current airway clearance technique physiotherapy service offered to patients with bronchiectasis?

Respiratory physiotherapy airway clearance techniques and services for bronchiectasis

Please answer these questions based on your usual circumstances/service.

9. Please use the comments box below to tell us anything else you think is important about airway clearance techniques and physiotherapy services for bronchiectasis:

Respiratory physiotherapy airway clearance techniques and services for bronchiectasis

* 10. The Coronavirus situation may have caused changes to your current practice and service delivery for people with bronchiectasis. Please use the box below to tell us if your service has changed (or not), how it has changed and if any of these changes are likely to continue in the future.

Respiratory physiotherapy airway clearance techniques and services for bronchiectasis

* 11. I wish to be entered into the iPad prize draw for completing this survey.

If yes, Queen's University Belfast will require your name and address, or email address to facilitate the iPad prize draw. Please click here to provide your details (this will open a new page, please return to this page when completed).

◯ Yes. I have provided my details.

◯ No

QUEEN'S UNIVERSITY BELFAST

Respiratory physiotherapy airway clearance techniques and services for bronchiectasis

SURVEY COMPLETE

Thank you for completing this survey.

If you would like more information about this survey research, please contact the Principal Investigators: Prof Judy Bradley or Dr Katherine O'Neill on ACTBEstudy@qub.ac.uk /00 44 (0)28 9097 6005.
