# Supplementary material for: Digital technologies in bronchiectasis physiotherapy services: a survey of patients and physiotherapists in a UK centre
Source: ERJ Open Res. 2024 Oct 6;10(3):00013-2024. doi: 10.1183/23120541.00013-2024 (PMC11163277; doi:10.1183/23120541.00013-2024)
Supplement: Supplementary file 2 [file 00013-2024.SUPPLEMENT2.pdf]

QUEEN'S UNIVERSITY BELFAST

Airway Clearance Techniques (chest physiotherapy) for people with Bronchiectasis

Version 2 14/10/2019

About the Study

You are being invited to participate in this survey research as you took part in the EMBARC (THE EUROPEAN BRONCHIECTASIS REGISTRY) study or the Bronch UK study and indicated that you would be willing to be contacted for future research. This study is being led by Queen's University Belfast (Dr Katherine O'Neill, Prof Judy Bradley) and funded by the Northern Ireland Chest Heart and Stroke.

What is this survey about?
Bronchiectasis leads to a build-up of mucus which can make infections more likely and also causes worsening of symptoms of cough and shortness of breath. Therefore, clearing the airways of mucus effectively is very important. Airway Clearance Techniques (chest physiotherapy) are exercises that help you to remove mucus from the lungs and cough it out. The purpose of this survey is to ask you about the physiotherapy services which deliver Airway Clearance Techniques in bronchiectasis.

Who should complete this survey?
Please complete this survey if you are an adult who has a diagnosis of bronchiectasis.

Do I have to take part?
Your participation in this survey research is entirely voluntary. It is up to you to decide whether or not you take part. If you participate, you can withdraw at any time by closing the page in the survey.

What will happen if I take part?
Taking part involves completing this survey which will take approximately 20 minutes. The survey questions will ask you about your experience using Airway Clearance Techniques and physiotherapy services for your bronchiectasis. You will also be asked to complete the survey a second time, after completing the first survey.

What are the possible benefits?
Whilst there will be no direct health benefits for you by participating in this survey, we hope to use the findings to help improve physiotherapy services for people with bronchiectasis. People with bronchiectasis have said that access to chest physiotherapy is a top research priority (EMBARC consensus statement). Your information and views are very important to us as it is you who really knows how services could be improved to help you and other people with bronchiectasis.

Confidentiality and Data Protection

All information collected during the course of this survey research will be kept strictly confidential, and will be stored securely at Queen's University Belfast. All data stored on the Queen's University Belfast system will comply with the University's policy. All information accessed from the EMBARC registry is anonymous and not identifiable to you. This survey has been the subject of ethical review and adheres to applicable data security standards. All data will be destroyed within 5 years after completion of the study. Data collected from the survey may be transferred to various locations where SurveyMonkey has offices and shared with other organisations.

Queen's University Belfast is the sponsor for this study based in the United Kingdom. We will be using information from you in order to undertake this study and will act as the data controller for this study. This means that we are responsible for looking after your information and using it properly. Queen's University Belfast will keep identifiable information about you for 5 years after the study has finished. Your rights to access, change or move your information are limited, as we need to manage your information in specific ways in order for the research to be reliable and accurate. If you withdraw from the study, we will keep the information about you that we have already obtained. To safeguard your rights, we will use the minimum personally identifiable information possible.
You can find out more about how we use your information.

Who can I contact if I have more questions?

You can contact the principal investigator (Prof Judy Bradley/Dr Katherine O'Neill) on ACTBEStudy@qub.ac.uk / 00 44 (0)28 9097 6005 if you have any questions or if you do not wish to be take part.

This survey consists of 2 sections:

Section 1: Consent
Sections 2: Questions

QUEEN'S UNIVERSITY BELFAST

Airway Clearance Techniques (chest physiotherapy) for people with Bronchiectasis

Section 1: Consent form

* 1. I understand that my participation in this survey research study is voluntary and that I am free to withdraw at any time by closing the survey page.

☐ Yes

* 2. I understand that anonymized data gathered in this study will be used in research publications.

☐ Yes

* 3. I understand that data collected during the study may be looked at by individuals involved in the study or from regulatory authorities, where it is relevant to my participation in this research.

☐ Yes

* 4. I understand that the data collected will be stored in a safe manner in Queen's University Belfast and will be destroyed 5 years after completion of the study.

☐ Yes

* 5. I understand that I need to provide my unique study ID (as provided on my invitation) in order to link my survey responses to my anonymous clinical data from the EMBARC registry.

☐ Yes

* Please enter your unique study ID as provided on the invitation letter:

If you so not have your unique study ID please contact the research team on 0044 (0)28 9097 6005 or ACTBEStudy@qub.ac.uk

* **6. (Optional)** I agree to complete the survey a second time, 12 months from now. I am providing my contact email or home address so that the survey can be sent directly to me, by the Queen's University Belfast research team.

My details will only be used for this purpose. If you agree, please click here to provide your contact email or home address (this will open a new page, please return to this page once completed).

- [ ] Yes, I have provided my contact details
- [ ] No

* **7. (Optional)** I am willing to be contacted by the research team to help interpret the overall survey results.

If you agree, please click here to provide your contact email or home address (this will open a new page, please return to this page once completed).

- [ ] Yes, I have provided my contact details
- [ ] No

QUEEN'S UNIVERSITY BELFAST

Airway Clearance Techniques (chest physiotherapy) for people with Bronchiectasis

Section 2: Questions on your Airway Clearance Technique practice

* 1. Think about the **first time** you found out about airway clearance techniques.

How were you **first** taught airway clearance techniques for your bronchiectasis?

Please select only one.

- ◯ By a physiotherapist whilst an outpatient at a hospital clinic appointment
- ◯ By a physiotherapist whilst an in-patient at a hospital
- ◯ By a physiotherapist in a health centre in the community
- ◯ By a physiotherapist in my home
- ◯ Through a support group
- ◯ Through someone else with bronchiectasis
- ◯ I found out on my own
- ◯ Don't know/ don't recall
- ◯ I have never been taught airway clearance techniques
- ◯ Other (please specify)

Airway Clearance Techniques (chest physiotherapy) for people with Bronchiectasis

* 2. Think about the **first visit** when you saw a physiotherapist about airway clearance techniques for your bronchiectasis.

Think about **who you see and where you are seen** for airway clearance techniques for your bronchiectasis.

What do you think is most important for a **first visit** for airway clearance techniques?

Please rank in order of importance, **1 being most important, 4 being least important.**

- Seeing a physiotherapist who is a specialist in bronchiectasis
- Seeing a physiotherapist who works with respiratory patients (but is not a necessarily specialist in bronchiectasis)
- Having the visit in a dedicated bronchiectasis clinic
- Having the visit in a location of my choice

Airway Clearance Techniques (chest physiotherapy) for people with Bronchiectasis

* 3. Think about the **first visit** when you saw a physiotherapist about airway clearance techniques for your bronchiectasis.

What do you think are the most important parts of the **content of a first visit** with a physiotherapist for airway clearance techniques for your bronchiectasis?

Please rank in order of importance, **1 being most important, 8 being least important.**

- Receiving information about the importance of airway clearance techniques
- A physiotherapist performing an assessment of my chest
- Being taught to do an airway clearance technique
- Setting a personal action plan for my airway clearance techniques (an action plan is a written plan that details your symptoms and what to do when well and when not well)
- A physiotherapist having enough time for my first appointment
- Receiving information about other physiotherapy treatments in addition to airway clearance techniques (for example; pulmonary rehabilitation, medications for airways clearance, incontinence treatments)
- Receiving information about support groups
- Receiving contact details and instruction on how to access a physiotherapist in the future

QUEEN'S UNIVERSITY BELFAST

Airway Clearance Techniques (chest physiotherapy) for people with Bronchiectasis

* 4. What parts of your bronchiectasis condition do you think airway clearance techniques help with?

Please select all that apply.

- [ ] Preventing a chest infection
- [ ] Managing a chest infection
- [ ] Sticky sputum
- [ ] Excess sputum
- [ ] Persistent and increased cough
- [ ] Ineffective cough
- [ ] Shortness of breath
- [ ] Other, please detail

QUEEN'S UNIVERSITY BELFAST

Airway Clearance Techniques (chest physiotherapy) for people with Bronchiectasis

* 5. What type/s of airway clearance techniques do you use when you are well? You may use more than one.

Please select all that apply.

- [ ] None
- [ ] Postural Drainage
- [ ] Active Cycle of Breathing Techniques (cycle of deep breaths, breathing control, huff and cough)
- [ ] Autogenic Drainage
- [ ] Clapping/Percussion
- [ ] Huff (forced expiration technique)
- [ ] Cough (please select if you use cough as part of other techniques)
- [ ] Positive Expiratory Pressure Mask
- [ ] Acapella device
- [ ] Flutter device
- [ ] Aerobika device
- [ ] High Frequency Chest Wall Oscillation device
- [ ] ELTGOL (Efficacité de l'expiration lente totale glotte ouverte en décubitus láteral / Slow expiration with the glottis opened in the lateral posture
- [ ] Exercise and/or physical activity
- [ ] Non-invasive ventilation device
- [ ] Cough assist device
- [ ] Intrapulmonary Positive Pressure / Bird device
- [ ] Other (please specify)

QUEEN'S UNIVERSITY BELFAST

Airway Clearance Techniques (chest physiotherapy) for people with Bronchiectasis

* 6. Do you **change the type/s** of airway clearance technique you use **when you have ar increase in symptoms or are unwell with a chest infection?**

- ◯ No, I do not change the type/s of airway clearance technique(s) when I have an increase in symptoms or are unwell with a chest infection.
- ◯ Yes, I change the type/s of airway clearance technique(s) when I have an increase in symptoms or are unwell with a chest infection.

QUEEN'S UNIVERSITY BELFAST

Airway Clearance Techniques (chest physiotherapy) for people with Bronchiectasis

* 7. How **often** do you do airway clearance techniques when you are well?

Please select only one.

- ◯ More than twice daily
- ◯ Twice daily
- ◯ Daily
- ◯ 2 times per week or more
- ◯ Weekly
- ◯ Monthly
- ◯ Other (please state how often)

Airway Clearance Techniques (chest physiotherapy) for people with Bronchiectasis

* 8. Do you **change how often** you do your airway clearance techniques when you have an **increase in symptoms or are unwell with a chest infection?**

- ○ No, I do not change how often I do my airway clearance technique(s) when I have an increase in symptoms or when I am unwell with a chest infection.
- ○ Yes, I change how often I do my airway technique(s) when I have an increase in symptoms or when I am unwell with a chest infection.

Airway Clearance Techniques (chest physiotherapy) for people with Bronchiectasis

* 9. **How long**, on average, does each airway clearance session last when you are well?

Please select one.

- ◯ Less than 10 minutes
- ◯ 10 minutes
- ◯ Greater than 10 minutes

Airway Clearance Techniques (chest physiotherapy) for people with Bronchiectasis

* 10. Do you **change how long** you do your airway clearance techniques when you have an **increase in symptoms or are unwell with a chest infection?**

- ◯ No, I do not change how long I do my airway clearance technique(s) when I have an increase in symptoms or when I am unwell with a chest infection.
- ◯ Yes, I change how long I do my airway clearance technique(s) when I have an increase in symptoms or when I am unwell with a chest infection.

QUEEN'S UNIVERSITY BELFAST

Airway Clearance Techniques (chest physiotherapy) for people with Bronchiectasis

* 11. What is the **main thing** that guides how often and how long you do your airway clearance technique session?

Please select one.

- ◯ What the physiotherapist told me to do
- ◯ A set length of time that I decide
- ◯ Depending on how much sputum I have
- ◯ Until I feel that my chest is clear
- ◯ Until I feel too tired/fatigued to continue
- ◯ Until another symptom stops me from continuing e.g. wheeze, shortness of breath, light head
- ◯ Other, please detail

QUEEN'S UNIVERSITY BELFAST

Airway Clearance Techniques (chest physiotherapy) for people with Bronchiectasis

* 12. How do you currently receive **follow-up** for your airway clearance techniques?

Follow-up is when you see the physiotherapist again.

Please **select all that apply.**

- [ ] I have not been followed-up
- [ ] I am followed-up by a physiotherapist at hospital
- [ ] I am followed-up by a physiotherapist at a community health centre
- [ ] I am followed-up by a physiotherapist in my home
- [ ] I receive telephone follow-up from a physiotherapist
- [ ] Other, please detail

QUEEN'S UNIVERSITY BELFAST

Airway Clearance Techniques (chest physiotherapy) for people with Bronchiectasis

* 13. Think about **who you see and where you are seen** for airway clearance techniques for your bronchiectasis.

What do you think is most important for a **first follow-up** for airway clearance techniques?

Please rank in order of importance, **1 being most important, 4 being least important.**

- Seeing a physiotherapist who is a specialist in bronchiectasis
- Seeing a physiotherapist who works with respiratory patients (but is not a necessarily specialist in bronchiectasis)
- Having the visit in a dedicated bronchiectasis clinic
- Having the visit in a location of my choice

QUEEN'S UNIVERSITY BELFAST

Airway Clearance Techniques (chest physiotherapy) for people with Bronchiectasis

* 14. As the physiotherapy service for people with bronchiectasis is modernised, there may be new ways for a physiotherapist to follow you up for your airway clearance techniques.

Which of following do you think you would use?

Please select all that apply.

- [ ] Access to the physiotherapist by phone
- [ ] Use of a website and/or phone app to see a physiotherapist (e.g. Skype)
- [ ] Use of a website and/or phone app to collect and share information about your condition (e.g. information about your symptoms, lung function) and communicate with a physiotherapist
- [ ] Use of a website and/or phone app to help you decide which airway clearance techniques to use
- [ ] None
- [ ] Other, please detail

QUEEN'S UNIVERSITY BELFAST

Airway Clearance Techniques (chest physiotherapy) for people with Bronchiectasis

* 15. Do you currently take any medication to help you with your airway clearance (for example, carbocisteine, hypertonic saline, isotonic saline, Dnase, Mannitol, bronchodilators)?

- ◯ Yes
- ◯ No

QUEEN'S UNIVERSITY BELFAST

Airway Clearance Techniques (chest physiotherapy) for people with Bronchiectasis

* 16. Which medication do you currently take to help you with your airway clearance?

Please select all that apply.

- [ ] Carbocisteine (for example, Mucodyne)
- [ ] Hypertonic Saline (for example, 3%, 5% or 7% hypertonic saline)
- [ ] Isotonic Saline (for example, 0.9% isotonic saline)
- [ ] DNase (for example, Pulmozyme)
- [ ] Mannitol
- [ ] Bronchodilator (for example, Short acting bronchodilators, Long acting bronchodilators, combination bronchodilators)
- [ ] Other

QUEEN'S UNIVERSITY BELFAST

Airway Clearance Techniques (chest physiotherapy) for people with Bronchiectasis

* 17. How do you time this medication with your current airway clearance technique?

	I take it **before** my airway clearance technique	I take it **after** my airway clearance technique	I take it **during** my airway clearance technique	I **don't** time this medication around my airway clearance technique
Carbocisteine (for example, Mucodyne)	○	○	○	○
Hypertonic Saline (for example, 3%, 5% or 7% hypertonic saline)	○	○	○	○
Isotonic Saline (for example, 0.9% isotonic saline)	○	○	○	○
DNase (for example, Pulmozyme)	○	○	○	○
Mannitol	○	○	○	○
Bronchodilator (for example, Short acting bronchodilators, Long acting bronchodilators, combination bronchodilators)	○	○	○	○
Other	○	○	○	○

QUEEN'S UNIVERSITY BELFAST

Airway Clearance Techniques (chest physiotherapy) for people with Bronchiectasis

* 18. How do you think we could improve the current airways clearance technique physiotherapy service offered to people with bronchiectasis?

Please provide your comments:

* 19. Please use the comments box below to tell us anything else you think is important about airway clearance techniques and physiotherapy services for your bronchiectasis.

* 20. I wish to be entered into the iPad prize draw for completing this survey.

If yes, Queen's University Belfast will require your name and address, or email address to facilitate the iPad prize draw. Please click here to provide your details (this will open a new page, please return to this page once completed).

- ◯ Yes. I have provided my details.
- ◯ No

Airway Clearance Techniques (chest physiotherapy) for people with Bronchiectasis

SURVEY COMPLETE

Thank you for completing this survey.

If you would like more information about bronchiectasis or airway clearance techniques, please contact Prof Judy Bradley or Dr Katherine O'Neill on ACTBEstudy@qub.ac.uk / 00 44 (0)28 9097 6005
